# Comparison of cement pressurisation in flanged and unflanged acetabular cups

**DOI:** 10.1186/1749-799X-7-5

**Published:** 2012-02-03

**Authors:** Rajarshi Bhattacharya, Fahad G Attar, Sarah Green, Andrew Port

**Affiliations:** 1 Department of Trauma and Orthopaedic Surgery, Imperial College Healthcare NHS Trust, London, UK; 2 Department of Trauma and Orthopaedic Surgery, James Cook University Hospital, Middlesbrough, TS4 3BW, UK; 3 School of Engineering, Durham University, DH1 3HP, UK

## Abstract

**Background:**

This biomechanical study examined difference in cement pressures generated by flanged and unflanged acetabular cups in hip arthroplasty.

**Method:**

Using a model acetabulum, cement was inserted and pressurised followed by cup insertion and pressurisation. Pressures were recorded using transducers in the acetabulum. We compared Charnley Ogee (flanged), Exeter contemporary (flanged) and Exeter low profile (unflanged) cups using Simplex and CMW1 cements in turn.

**Results:**

Using Simplex, Charnley Ogee cup generated highest initial peak pressure and overall mean pressure. Exeter unflanged cup generated higher initial and mean pressures compared to Exeter flanged cup. With CMW, there was no significant difference between the pressures generated by the cups.

**Conclusions:**

Our experiment suggests that flanged cups do not consistently generate significantly higher cement pressures compared to unflanged cups.

## Background

Aseptic loosening of the acetabular component is widely regarded as the commonest cause for revision surgery in THR [1-4]. Cement pressurisation has been shown to improve cup fixation in the acetabulum and in this regard, sustained pressurisation throughout the period of cement polymerisation has been shown to be more important than peak pressures [5,6]. Cement pressurisation prior to cup insertion is regarded as one of the most important factors in affecting the quality of cementation and thereby the survival of the cup. But once the cup is inserted, it would seem logical that allowing the cement to be contained inside rather than being extruded out would also improve cementation. In order to achieve this, flanged cups were introduced in 1976 [7], with a view to improve fixation. The idea was that a well fitting flange seated just inside the rim of the acetabulum would allow better containment of cement thereby generating higher cement pressures. However, the precise usefulness of a flange in practical situations has been repeatedly questioned.

The debate between flange and unflanged cups in Total Hip Arthroplasty has been going on for quite some time now. A few studies have attempted to compare in vitro the pressures generated by flanged and unflanged cups but most of them involve the peak pressures only and not the mean pressures generated throughout the entire period of cement polymerisation. We felt that the mean pressure generated was more important in this regard and hence this study was set up.

## Methods

**Figure 1:**
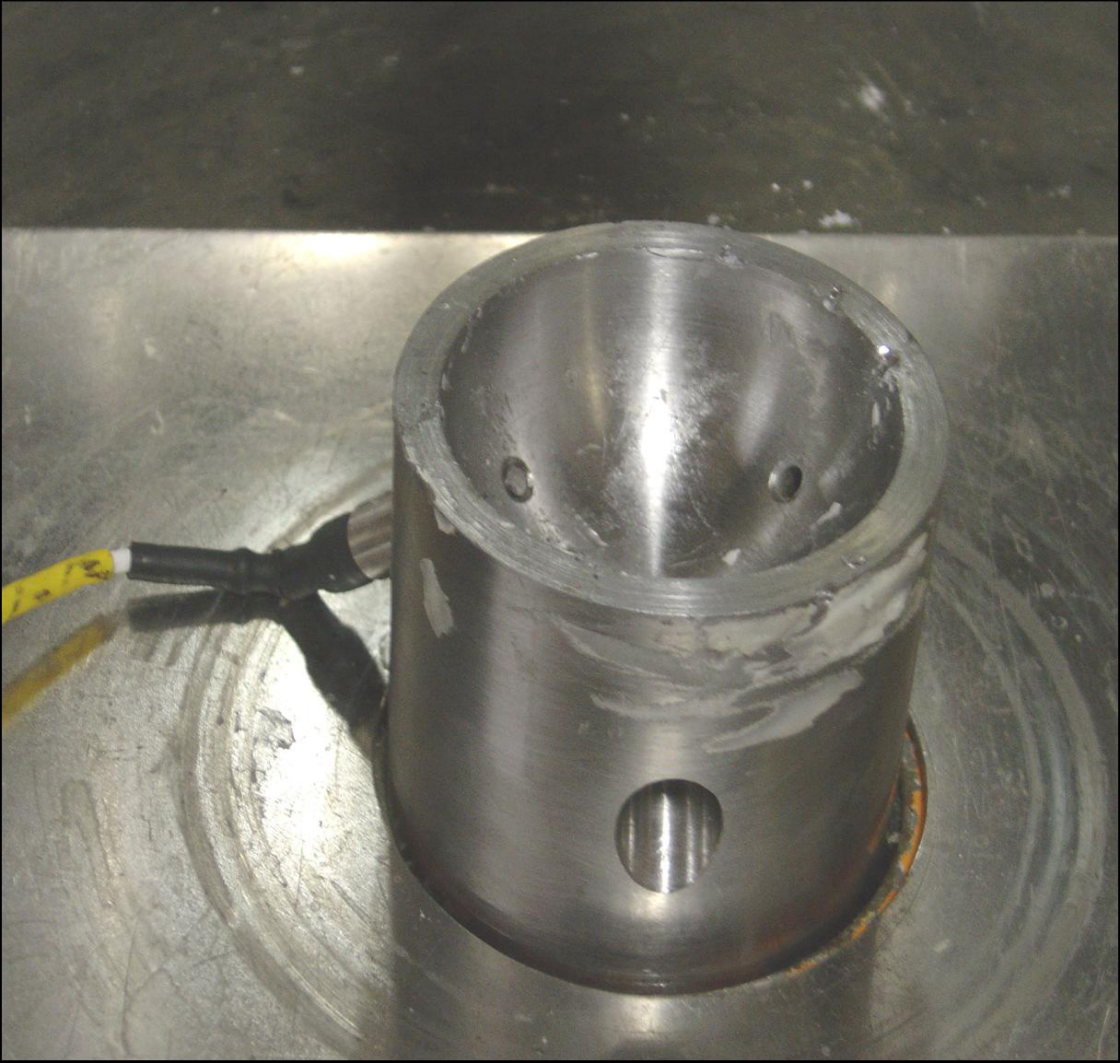
**Metal acetabulum with a transducer**.

**Figure 2:**
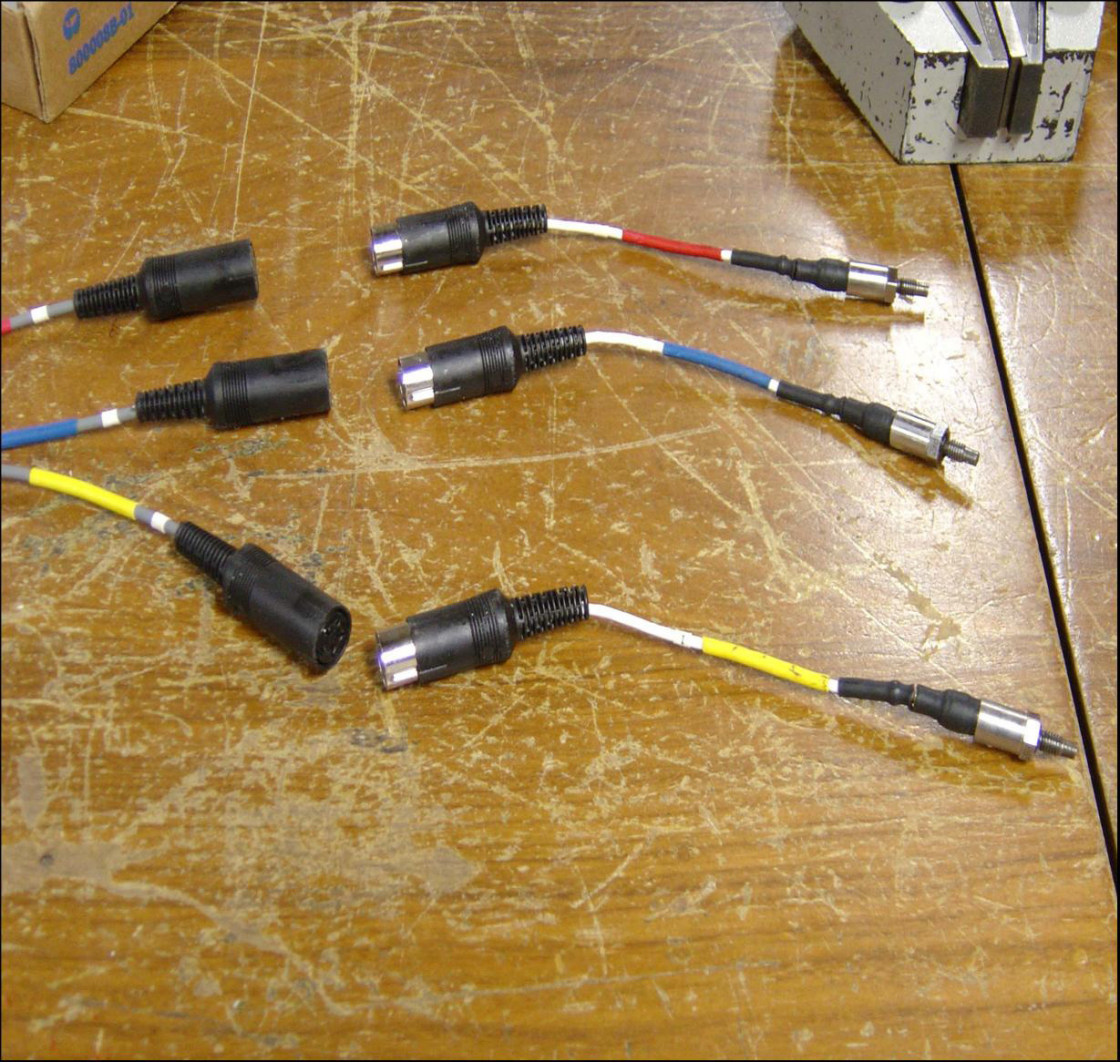
**Pressure transducers**.

We performed a biomechanical test simulating clinical situation as far as was practicable. A metal acetabulum model was created with an inside diameter of 52 mm (Figure 1). It had 3 holes equidistant from each other, with threads cut in the holes where 3 diaphragmatic type piezoelectric pressure transducers (Figure 2) were screwed in. The tips of the transducers were flush with the surface of the acetabulum. The other end of each transducer was connected to a separate lead which all fed into a computer with software to generate the pressure-time graphs. The transducers were colour coded to correspond with the colours of the curves made by each of the 3 transducers.

3 different cup types were compared, with similar outer diameters of around 48 mm to ensure a uniform cement mantle of at least 2 mm all around. These were, the Charnley Ogee flanged cup (size 47 mm with an outer diameter of 47 mm selected as 48 mm cup is not produced by the manufacturers), the Exeter low profile cup (size 48 with an outer diameter 48 mm selected) which was unflanged, and the Exeter contemporary cup (size 52 mm cup which corresponds with an outer diameter of 48 mm) which was flanged with PMMA beads on the outer surface of the cup designed to prevent bottoming out of the cups. These sizes were chosen to be representative of the cup sizes that would normally be used in actual clinical situation for an acetabulum size of 52 mm, which was the size of our model acetabulum. The Charnley Ogee and the Exeter low profile are currently two of the most commonly used cups in hip arthroplasty, representative of the flanged and unflanged varieties respectively and hence these two were used for the comparison. The Exeter flanged cup is a relatively new product which claims to have additional benefits with the inclusion of beads to prevent bottoming out of the cup and a firmer flange compared to the Charnley cup to prevent eversion of the flange and consequent extrusion of cement during cup insertion and pressurisation. We wanted to observe whether these changes to the Exeter contemporary cup resulted in better pressure generation.

3 sets of experiment were performed using each cup type with Simplex cement giving a total of 9 readings. The whole experiment was then repeated using CMW1 cement. Simplex and CMW1 are the 2 types of cement used commonly in clinical practice in our hospital. We wanted to ensure that our readings were not influenced by cement properties and hence the experiment was performed using both types.

**Figure 3:**
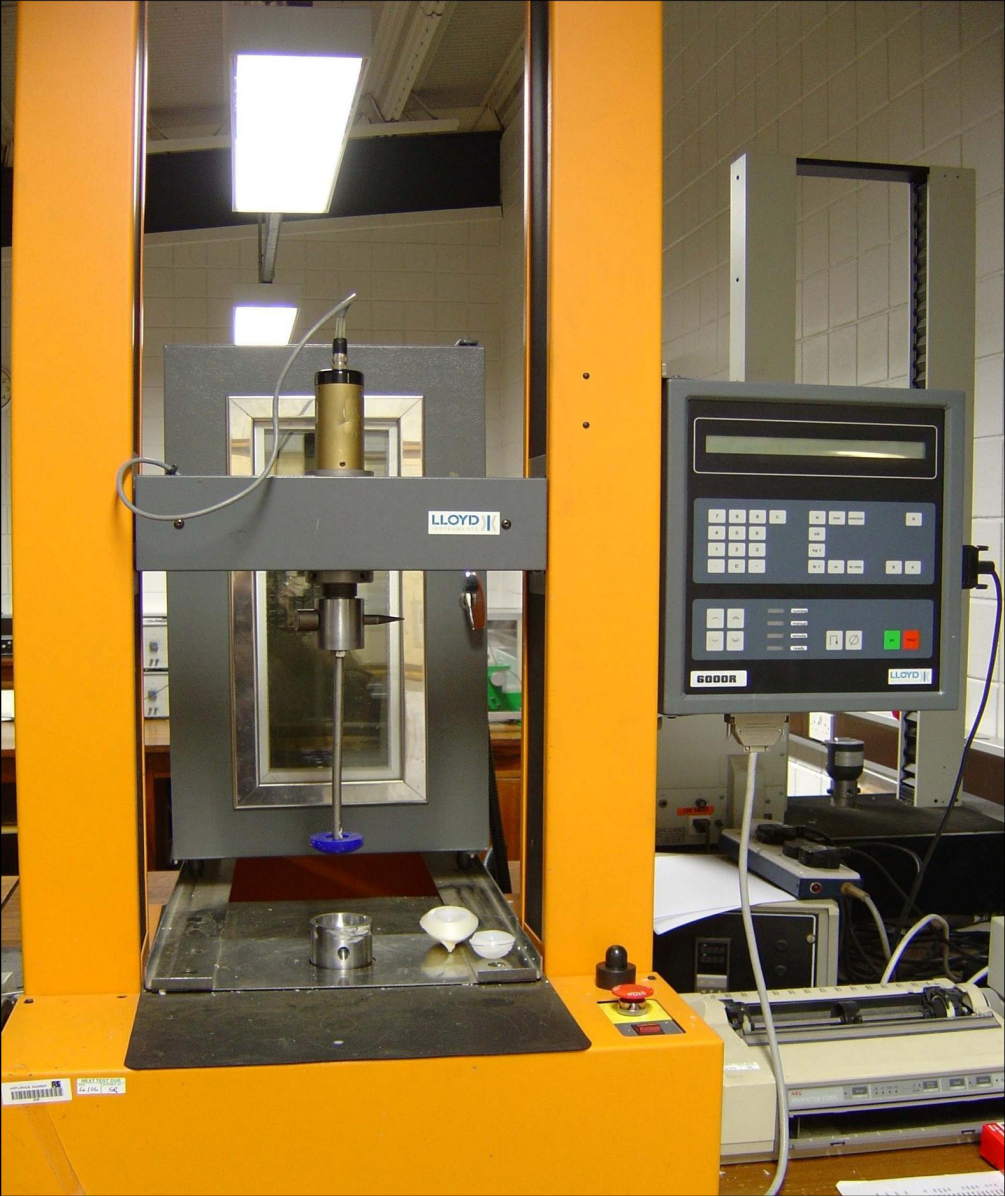
**Experimental set up**.

**Figure 4:**
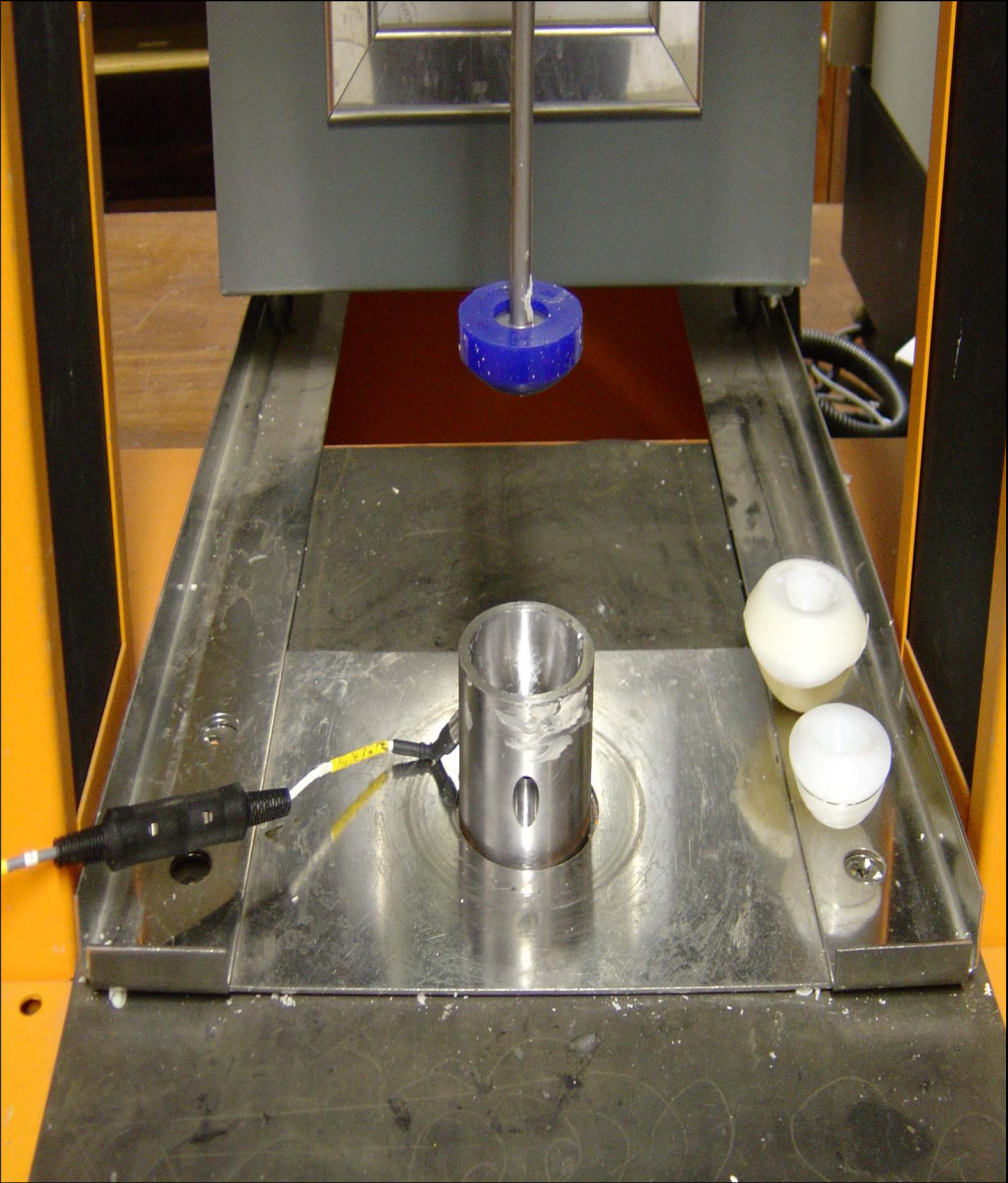
**experimental set up with transducer**.

A Lloyd Instruments 6000R plus universal testing machine was used to generate a static load of 70 N for the purpose of the initial cement pressurisation as well as subsequent cup insertion and pressurisation (Figures 3 and 4). A series of pilot readings were obtained whereby an arthroplasty surgeon manually cemented the cups using the set up and 70 N was found to be the force that could be comfortably maintained throughout the process of cement polymerisation without bottoming out of the cups. This pressure figure was hence chosen for our experiment. Besides, previous similar studies had also come up with similar figures for cement pressurisation [7,8].

The temperature of the lab was maintained at a uniform 20 degrees throughout the experiment. The cement was vacuum mixed according to the manufacturers' instructions (45 seconds for both cement types), and inserted into the metal acetabulum. The cement was then pressurised, until cup insertion, by a cement pressuriser fitted onto the universal testing machine. The acetabular cup was then inserted and pressurised using a cup pressuriser mounted on the universal testing machine. In the case of Simplex, the cup was inserted at 5 and 1/2 minutes and in the case of CMW the cup was inserted at 4 and 1/2 minutes (as per manufacturers' recommendations and clinical protocol), due to the increased viscosity of the CMW1. After each experiment, the residual cement was cleaned out from the acetabular model.

Continuous pressure recordings were obtained with each of the 3 pressure transducers simultaneously and these were represented both graphically on a pressure time curve as well as digitally at 10 seconds intervals till the end of cement polymerisation which was identified as the point where the pressure graphs plateaued near the baseline pressure and this corresponded to the expected in-vivo polymerization times for the Simplex and Palacos cements. This also corresponded to the time that a sample of extruded cement from the experiment had turned hard. The data was analysed using the *SPSS 11.0 *(Statistical Package for the Social Sciences, SPSS Inc., Chicago, Illinois). The pressures generated by the cups were compared within each cement group and not between cement groups, in order to prevent multiple variables from affecting the readings. A Kolmogorov-Smirnov test was initially used and this confirmed that the data was normally distributed. Independent sample t-tests were then performed within each cement sub-group comparing two types of cup at a time. As the sample sizes were small we also calculated the median and the interquartile range.

## Results

The detailed results are summarised in Tables 1 and 2.

**Table 1:** Comparison table for peak and mean pressures.

	Charnley Ogee (flanged)	Exeter Contemporary (flanged)	Exeter low profile (unflanged)
With simplex			

Peak pressure	*53.7 (3.6) (55.5 +/- 6.4)*	*17.9 (11.6) (13.9 +/- 2.2)*	*34.1 (3.6) (35.4 +/- 6.8)*

Mean pressure	*14.8 (9.5) (11.8 +/- 4.3)*	*4.2 (9.3) (1.2 +/- 0.9)*	*7.7 (7.0) (5.3 +/- 0.1)*

With CMW			

Peak pressure	*26.0 (1.0) (25.6 +/- 1.9)*	*12.9 (2.2) (11.9 +/- 4.0)*	*41.1 (5.8) (40.6 +/- 11.5)*

Mean pressure	*6.4 (7.5) (3.6 +/- 3.9)*	*4.5 (2.7) (3.7 +/- 0.9)*	*8.2 (5.0) (6.5 +/- 3.4)*

Values are Mean (SD) and (Median +/- Interquartile range) of the pressures in N/cm^2 ^

**Table 2:** Summary of Results

	Results	P values
Simplex		

**Peak pressure**	Charnley > Exeter unflanged	significant (p < 0.01)

	Charnley > Exeter flanged	significant (p < 0.01)

	Exeter unflanged > Exeter flanged	not significant

		

**Mean pressure**	Charnley > Exeter unflanged	not significant

	Charnley > Exeter flanged	significant (p < 0.05)

	Exeter unflanged > Exeter flanged	not significant

		

CMW		

**Peak pressure**	Charnley < Exeter unflanged	significant (p < 0.05)

	Charnley > Exeter flanged	significant (p < 0.01)

	Exeter unflanged > Exeter flanged	significant (p < 0.01)

		

**Mean pressure**	Charnley < Exeter unflanged	not significant

	Charnley > Exeter flanged	not significant

	Exeter unflanged > Exeter flanged	not significant

When the experiment was performed using the Simplex cement, the Charnley flanged cup generated significantly higher peak pressures compared to the Exeter flanged and Exeter unflanged cups. The Exeter unflanged cup generated a higher peak pressure compared to the Exeter flanged cup although this difference was not statistically significant. When the mean pressures were compared, there was no significant difference between the Charnley (flanged) and the Exeter unflanged cup although the Charnley produced a significantly higher mean pressure compared to the Exeter flanged cup. The pressures generated between the two Exeter cup types were not significantly different. Thus with the simplex, although the Charnley cup generated significantly higher peak pressures, the effect of the flange on the mean pressure was not as pronounced and the Exeter flanged cup seemed to generate the least amount of pressurisation among the 3 cup types.

When the whole experiment was repeated using the CMW cement, the results were even more inconsistent, with the Exeter unflanged cup outperforming the Charnley in peak pressure generation and there being no significant difference between the 3 cups in mean pressure generation. However, here also, the Exeter flanged cup seemed to generate the least amount of cement pressure.

Looking at the results of the experiments closely it becomes quite obvious that the flanged cups do not seem to generate consistently higher cement pressures compare to unflanged cups and the Exeter flanged cup seems to fare the worst. If we exclude the Exeter flanged cup and compare the two most commonly used flanged and unflanged cups, the results still show that there isn't a great difference in cement pressurisation between the 2 cup types.

## Discussion

The debate between the superiority of cement pressurisation using flanged and unflanged cups is a hard one to resolve using the results of a single experiment. Proponents of the flanged cup claim that the flange helps to contain the cement better and hence helps in better pressurisation. The most important factor in effective cementation of the acetabular component is widely recognised to be the period of pre-cup insertion pressurisation. Hence the actual pressure generation during cup insertion is probably not hugely relevant.

A few experiments conducted earlier have tried to confirm the theoretical advantages of flanged cups in controlled laboratory settings. Oh et al, [9] compared pressurisation between flanged cups, unflanged cups and flanged cups with scalloped ends where all the cups were sunk to a predetermined depth. They concluded that flanged cups generated higher peak pressures and cement intrusion pressures. One of the problems with their study was that considerably higher loading pressures were required to sink the flanged cups to the predetermined levels, up to 10 times that of the loading pressure required for the other cup types. Shelly and Wroblewski [7] compared flanged and unflanged cups by using a standard loading pressure of 80 N and again concluded that flanged cups generated higher peak pressure and cement intrusion pressure. However, the above authors failed to comment on the sustained mean pressure generated during the phase of cement polymerisation.

Parsch et al [8] recently conducted a similar experiment using paired human acetabula and they looked at both peak and mean pressures and found that although flanged cups cause increase in peak pressures, there was no difference in mean pressures generated. Our results also confirm their findings.

One criticism of not sinking cups to predetermined depths during the experiments is that due to the mechanical nature of loading, there may be no indication of the cup bottoming out completely. However, this can be addressed in two ways. Firstly the loading pressure of 70 N that was selected is normally used in clinical practice in the clinical situation. It is not the maximum pressure that can be generated by a surgeon but rather a realistic and optimal pressure that is used to ensure a reasonable cement mantle and this was confirmed at the start of our experiment using pilot readings. This fact is also borne out by the other previous experiment looking at sustained cement pressure [8]. Besides, as the outer surface of the cups were not smooth, if the cups were to bottom out completely, the transducers would be in direct contact with the irregular outer surface of the cups which would cause an irregular haphazard reading as opposed to a smooth pressure curve. Indeed when the cups were taken out in our experiment there was a reasonable cement mantle at the bottom albeit not of the same uniform thickness as that of the sides.

A common criticism of flanged cups is that by the time the surgeon has finished preparing the shape of the cup, most of the flange has actually been cut off from the cup. Not to mention the existent debate about whether the flange should sit flush inside the rim or just cover the rim of the acetabulum. We feel that these issues can be resolved by not using flanged cups at all.

## Conclusions

The addition of flanges certainly adds to the operating time and inconvenience, due to the peri-operative trimming and templating of the cup involved. The theoretical advantages of flanged cups do not seem to be consistently borne out by the results of laboratory experiments; and no long term survival study has managed to show better or improved survival of flanged cups over unflanged ones. As a result we feel that flanged cups are not necessary to improve the cementation and hence survival of the acetabular component in Total Hip Arthroplasty.

## Competing interests

The authors declare that they have no competing interests.

## Authors' contributions

RB was the Principal researcher. FGA was the assistant researcher in conducting the experiments. SG was the biomechanical experiments' supervisor at Durham laboratory.

AP was the clinical supervising consultant and senior author. All authors read and approved the final manuscript.
